# Menthol cigarette pricing at military and community retail outlets in the United States

**DOI:** 10.1186/1471-2458-12-731

**Published:** 2012-09-01

**Authors:** Walker SC Poston, Sara A Jahnke, Christopher K Haddock, Melissa L Hyder, Jennifer E Taylor, Harry A Lando, Christopher M Kaipust

**Affiliations:** 1 Institute for Biobehavioral Health Research, NDRI-MA, NDRI: National Development and Research Institutes, Inc, 1920 West 143rd Street, Suite 120, Leawood, KS 66224, USA; 2 Vanderbilt University Medical Center, 1920 West 143rd Street, Suite 120, Leawood, KS 66224, USA; 3 University of Minnesota, 1920 West 143rd Street, Suite 120, Leawood, KS 66224, USA

**Keywords:** Cigarettes, Menthol, Military

## Abstract

**Background:**

Cigarette prices at military exchanges historically have been discounted. DoD Instruction 1330.9 has mandated that prices be within 5% of the price offered in the local community since 2001. Because minorities are highly represented in the military, we determined whether menthol cigarette prices, the leading choice of African Americans, were compliant with the instruction.

**Methods:**

We collected, via telephone, menthol cigarette price data from 48 randomly selected US military installation exchanges and matched local area Walmarts. We collected prices after taxes to determine the cost to consumer. Newport was selected as the index brand for menthol cigarettes because it is the leading and second leading brand smoked by African Americans and by Hispanics, respectively and has the second overall highest market share in the US.

**Results:**

Smokers purchasing menthols at exchanges would realize average savings of 22.78%. There were no significant differences in savings based on military service (F = 1.850, p = 0.152) or US Census Division (F = 1.226, p = 0.311: data not shown). In addition, not a single exchange price was compliant with the DoD instruction.

**Conclusions:**

Newport menthol cigarettes at military exchanges cost substantially less than the nearest Walmart, with an average savings of 23%. Our findings demonstrate that menthol cigarettes are substantially discounted on military installations, in a manner similar to other cigarette prices, and that DoD Instruction 1330.09 is not enforced.

## Background

Increasing tobacco prices is one of the most effective methods for reducing smoking while discounts increase consumption [1-3]. In the 1980s, the Department of Defense (DoD) began the process of de-incentivizing smoking by increasing prices to be comparable to those in civilian markets [4]. DoD Instruction 1330.9 [5], first implemented in 2001, mandated that tobacco products sold in military exchanges be “no lower than 5 percent below the most competitive commercial prices in the local community” [5]. Section 4.2.3. states that the intent of DoD Instruction 1330.9 is to "communicate to Service members that tobacco use is detrimental to health and readiness" [5]. However, it recently was demonstrated that the instruction is not effectively enforced for the most popular brand of cigarettes in the US [6], with discounts for both Marlboros and exchanges’ lowest-cost cigarettes averaging 25.4% and 14.5%.respectively. Additionally, fewer than 5% of exchanges were compliant with the instruction [6].

Menthol cigarettes, particularly Newport brand, are African Americans’ preferred cigarettes and very popular among other minority groups such as Hispanics [7-9]. Also, it is clear that tobacco companies target-market menthol cigarettes to minorities, particularly African Americans [9-12] and that smoking menthols is associated with lower cessation rates for African Americans and may contribute to negative health outcomes [9,13]. Because minorities, particularly African Americans, are highly represented in the US military enlisted ranks [14], we were interested in whether military exchange prices for menthol cigarettes were compliant with DoD Instruction 1330.9 [5]. In addition, we were interested in whether the percent discount would be greater for menthols when compared to Marlboros and whether menthol cigarette prices would be lower than the costs documented for Marlboro cigarettes [6], since it has been demonstrated that menthol cigarette prices are lower in areas with higher proportions of minorities and the military has high minority representation [10,14]. Thus, we examined menthol cigarette prices at military exchanges compared to local market prices.

## Methods

The study was approved by the National Development and Research Institutes’ Institutional Review Board. In our prior study of military exchange cigarette pricing [6], we obtained contact information for all US-based Army, Air Force, Navy, and Marine Corps exchanges (N = 201) using official websites (http://www.aafes.org and http://www.mynavyexchange.com) to compare costs of Marlboro cigarettes, the leading brand in the US [7,8], at military and civilian retails outlets. For the present investigation, we randomly selected 25% of military installation exchanges nationally, stratified by each of the four services in the previous study [6], excluding Air/Army Guard and joint bases, to ensure all branches were sampled. We telephoned the main exchange on each installation and asked for the cost of one pack of Newport menthol cigarettes because tobacco pricing is consistent across military retail outlets at any installation [5].

We similarly queried the nearest Walmart store, which is one of the most popular retail chains in the US and it has a growing international presence and was listed as the leading business in the 2011 Fortune 500 (see http://money.cnn.com/magazines/fortune/global500/2011/snapshots/2255.html). Walmart served as the local community price because: 1) it provided consistent comparisons across installations; 2) Walmart’s reputation for competitive prices; 3) it is simple to locate stores (http://www.walmart.com/cservice/ca_storefinder.gsp); and 4) Walmart was used as a benchmark for military cigarette pricing in Congressional testimony [15].

We collected prices after taxes to determine the cost to consumer. Newport was selected as the index brand for menthol cigarettes because it is the leading and second leading brand smoked by African Americans and by Hispanics, respectively [7-9]. It also is the leading brand of menthols and the second in overall US market share [7-9]. Comparisons between Walmart and exchange prices were based on the equation:

(1) $$\text{Savings}=\;\left(\frac{\text{Walmart}\;\text{Price}-\text{Exchange}\;\text{Price}}{\text{Walmart}\;\text{Price}}\right)*100$$

Comparisons were conducted using 2-tailed ANOVA with SPSSv19.

## Results

There were 48 matched Exchange/Walmart comparisons. Exchanges were distributed across the services, with 29.2% Air Force, 22.9% Army, 37.5% Navy, and 10.4% Marine Corps. Average distance between exchanges and Walmarts was 5.6 miles (SE = 0.83) and there were no significant service differences in distances to Walmarts (F = 0.342, p = 0.795). Table 1 presents the exchange and corresponding Walmart costs for Newports, the cost differences, and percent savings to consumers when purchasing Newports at military exchanges.

**Table 1:** Costs per cigarette pack and percentage of savings at military exchange compared with nearest Walmart for Newport Menthol cigarettes

	**Primary Service Affiliation**				
**Cigarette Pricing**	**Air Force**	**Army**	**Marines**	**Navy**	**All Installations**
N	14	11	5	18	48
Newport pack cost at Walmart	7.33 (0.21)	7.42 (0.40)	6.34 (0.28)	7.20 (0.32)	7.20 (0.17)
Newport pack cost at military exchange	5.88 (0.22)	5.61 (0.35)	5.17 (0.14)	5.28 (0.17)	5.52 (0.13)
Difference	1.45 (0.17)	1.81 (0.29)	1.17 (0.19)	1.92 (0.20)	1.68 (0.12)
Military savings,%	19.79 (2.12)	23.87 (3.20)	18.15 (2.52)	25.73 (1.91)	22.78 (1.26)

Note: entries represent mean costs (dollars) at Walmart, the military exchange, the mean difference between Walmart and the military exchange, and the percent savings over Walmart compared to the corresponding Military Exchange (standard error of the mean in dollars).

*No significant differences between services (F = 1.850, p = 0.152) or US Census Division (F = 1.226, p = 0.311: data not shown).

Smokers purchasing Newport Menthols at exchanges would realize savings between 18.15%- 25.73% depending on the service, with savings across all services averaging 22.78%. There were no significant differences in savings based on military service (F = 1.850, p = 0.152) or US Census Geographic Divisions, i.e., Northwest, Midwest, South, and West [16] (F = 1.226, p = 0.311: data not shown). In addition, not a single exchange price was compliant with the DoD instruction.

## Discussion

Newport menthol cigarettes at military exchanges cost substantially less than the nearest Walmart, with an average discount of 23%, far in excess of the allowable 5% mandated in DoD Instruction 1330.9 [5]. None of the selected exchanges were compliant with DoD Instruction 1330.9 [5] on pricing. However, we did not find that either Walmarts (M ± SE; $7.20 ± 0.17) or military exchanges ($5.52 ± 0.13) prices for menthol cigarettes were lower than those documented for Marlboros, the leading brand of cigarettes in the US [7], at Walmart ($6.73 ± 0.09) or military exchanges ($4.99 ± 0.07) [6], and the discount rates were similar (i.e., the average discount for Marlboros was 25.4%) [6]. Thus, military exchanges discounted menthol cigarettes, which have been documented to be target-marked to African Americans [9-12], in a manner similar to cigarettes that are marketed to the general public. In addition, the average prices of menthol cigarettes at both types of retailers were higher than those documented for Marlboros [6], which is consistent with findings from previous studies [10].

Previous research has indicated that, among military policies, those that address tobacco control are unique in their lack of enforcement [17-20]. While the military often takes a very aggressive stance against products which are potentially detrimental to the health of military members, they often are arguably less harmful than cigarettes. For example, when anecdotal evidence suggested Ephedra may pose negative health risks, the military banned its use by personnel much earlier than similar restrictions in the civilian sector [20]. While the scientific literature clearly demonstrates a significant, negative impact on health and readiness from tobacco use, the US military continues to sell cigarettes at deep discounts and accommodate its use.

This study had a number of strengths including the random selection of military exchanges, the use of a standardized community comparison for cigarette pricing, and the representation of all major military service exchanges. For example, Walmart provided an ideal comparison for a national study of cigarette pricing on military installations [6]. Nonetheless, it is possible that cigarette prices lower than Walmart may be available in the community at specialty establishments such as smoke shops or discount tobacco outlets. However, conducting a comprehensive pricing search for all retail outlets in the community where all of the randomly selected military exchanges were located was not feasible. Another weakness is that DoD Instruction 1330.09 [5] does not define “the most competitive commercial prices in the local community.” A liberal reading of this regulation may allow exchanges to base their prices on discount tobacco outlets, or even other military exchanges, which sell cigarettes at prices deeply discounted even when compared to Walmart. However, when extensive efforts have been made to find the lowest cost comparison in a given community, rates at exchanges often are considerably lower than the retail outlet with the deepest discount in a community [21].

Military personnel correctly perceive that tobacco control policies are weakly enforced [17-20]. Given tobacco’s significant negative impact on health and readiness and its associated medical and productivity costs [22], the lack of enforcement of DoD Instruction 1330.09 [5] is troubling. Deeply discounted cigarette prices at exchanges do not communicate DoD Instruction 1330.09’s message that “tobacco use is detrimental to health and readiness” [5]. The current study, along with previous results [6], demonstrate that cigarettes are substantially discounted on military installations and that DoD Instruction 1330.09 [5] is not enforced. We found that most military tobacco control policy leaders and installation-level tobacco control managers believed that service and installation commanders would be supportive of increasing tobacco prices [20], so eliminating discounts, or at least enforcing the instruction, should be a priority. However, because Congress strongly influences pricing on military installations [4], it is unclear whether the health and wellness of service members will be made a priority over the politics of tobacco.

## Conclusions

Newport menthol cigarettes at military exchanges were substantially cheaper than the nearest Walmart, with an average cost savings of 23%. There were no significant differences in costs based on region of the country or military service branch. Our data demonstrate that cigarettes target marketed to ethnic minority groups can be purchased on military installations at substantially discounted prices. This finding is consistent with previous economic evaluations (e.g., [6]) and provides evidence that DoD Instruction 1330.09 does not eliminate pricing as an incentive to purchase tobacco at military retail. The failure of military regulation to effectively control the discounting of cigarettes likely contributes to high rates of tobacco use among active duty military members and veterans.

## Competing interests

The authors declare that they have no competing interests.

## Authors’ contributions

WSC Poston was involved in the study origination and design, obtaining funding, data analysis and interpretation, and was chiefly responsible for writing the manuscript. SA Jahnke was involved in the study origination and design, study supervision, data acquisition, and critical revision of the manuscript. CK Haddock was involved in the study origination and design, obtaining funding, data analysis and interpretation, and critical revision of the manuscript. M Hyder was involved in study supervision, data acquisition, and critical revision of the manuscript. JE Taylor was involved in the study origination and design and critical revision of the manuscript. HA Lando was involved in the study origination and design and critical revision of the manuscript. C Kaipust was involved in data acquisition, interpretation of results, and critical revision of the manuscript**.** All authors read and approved the final manuscript.

## Pre-publication history

The pre-publication history for this paper can be accessed here:

http://www.biomedcentral.com/1471-2458/12/731/prepub
